# Network visualization of genes involved in skeletal muscle myogenesis in livestock animals

**DOI:** 10.1186/s12864-024-10196-3

**Published:** 2024-03-19

**Authors:** Fatemeh Mohammadi Nejad, Mohammadreza Mohammadabadi, Zahra Roudbari, Abdolvahab Ebrahimpour Gorji, Tomasz Sadkowski

**Affiliations:** 1 Department of Animal Science, Faculty of Agriculture, Shahid Bahonar University of Kerman, Kerman, Iran; 2 Department of Animal Science, Faculty of Agriculture, University of Jiroft, Jiroft, Iran; 3 Department of Physiological Sciences, Faculty of Veterinary Medicine, Warsaw University of Life Sciences, Warsaw, Poland

**Keywords:** Skeletal muscle, Myogenesis, Hub genes, Livestock animals

## Abstract

**Background:**

Muscle growth post-birth relies on muscle fiber number and size. Myofibre number, metabolic and contractile capacities are established pre-birth during prenatal myogenesis. The aim of this study was to identify genes involved in skeletal muscle development in cattle, sheep, and pigs - livestock.

**Results:**

The cattle analysis showed significant differences in 5043 genes during the 135–280 dpc period. In sheep, 444 genes differed significantly during the 70–120 dpc period. Pigs had 905 significantly different genes for the 63–91 dpc period.The biological processes and KEGG pathway enrichment results in each species individually indicated that DEGs in cattle were significantly enriched in regulation of cell proliferation, cell division, focal adhesion, ECM-receptor interaction, and signaling pathways (PI3K-Akt, PPAR, MAPK, AMPK, Ras, Rap1); in sheep - positive regulation of fibroblast proliferation, negative regulation of endothelial cell proliferation, focal adhesion, ECM-receptor interaction, insulin resistance, and signaling pathways (PI3K-Akt, HIF-1, prolactin, Rap1, PPAR); in pigs - regulation of striated muscle tissue development, collagen fibril organization, positive regulation of insulin secretion, focal adhesion, ECM-receptor interaction, and signaling pathways (PPAR, FoxO, HIF-1, AMPK). Among the DEGs common for studied animal species, 45 common genes were identified. Based on these, a protein-protein interaction network was created and three significant modules critical for skeletal muscle myogenesis were found, with the most significant module A containing four recognized hub genes - *EGFR*, *VEGFA*, *CDH1*, and *CAV1*. Using the miRWALK and TF2DNA databases, miRNAs (bta-miR-2374 and bta-miR-744) and transcription factors (CEBPB, KLF15, RELA, ZNF143, ZBTB48, and REST) associated with hub genes were detected. Analysis of GO term and KEGG pathways showed that such processes are related to myogenesis and associated with module A: positive regulation of MAP kinase activity, vascular endothelial growth factor receptor, insulin-like growth factor binding, focal adhesion, and signaling pathways (PI3K-Akt, HIF-1, Rap1, Ras, MAPK).

**Conclusions:**

The identified genes, common to the prenatal developmental period of skeletal muscle in livestock, are critical for later muscle development, including its growth by hypertrophy. They regulate valuable economic characteristics. Enhancing and breeding animals according to the recognized genes seems essential for breeders to achieve superior gains in high-quality muscle mass.

**Supplementary Information:**

The online version contains supplementary material available at 10.1186/s12864-024-10196-3.

## Background

Skeletal muscle is a complex structure, which sonsists of muscle fibers, intramuscular fat, connective tissue, nerves and blood vessels, and is one of the main components of the musculoskeletal system, in consumer terms, a valuable food product - meat [1]. Livestock is of great economic importance worldwide [2]. Livestock are genetically improved with breeding applications that promote meat yield traits to maximise slaughter efficiency and produce meat of the quality desired by the customer [3].

Developing fetal skeletal muscles undergo two separate stages of myogenesis. After the appearance in the embryonic phase of the muscle myotubes (primary myogenesis), forming a scaffold for subsequent waves of myoblasts, secondary myogenesis occurs as a result of the proliferation and fusion of myoblasts in order to increase the number of myofibres (secondary myogenesis) [4]. Slower myogenesis rates and fewer myoblasts entering the cell cycle can have long-term consequences [5], as the major variables that influence skeletal muscle development are myoblast proliferation and differentiation [6]. Muscle growth potential is directly influenced by myoblast proliferation, differentiation and fiber hypertrophy [7], which processes are governed by myogenic and regulatory factors [6]. At the end of the fetal and neonatal period, some of the myogenic cells become inactive going into a quiscent state - satellite cells [8]. The number of myogenic precursor cells determines the population of muscle fibers, in livestock usually established prenatally, and the total number of satellite cells present in the muscle after birth. Since the muscle fiber population does not increase after birth, myogenesis during the prenatal period has a significant impact on the development and subsequent maturation of skeletal muscle [9]. Postnatally, satellite cells undergo activation and then fuse with pre-existing muscle fibers, resulting in muscle hypertrophy [8]. Given that skeletal muscle development at the prenatal stage is crucial for growth, hypertrophy, maturation and therefore the quantity and quality of meat produced, the identification of gene networks involved in livestock myogenesis is very important. The aim of the current research was to find the hub genes and their networks key and common to the process of myogenesis in cattle, sheep and pigs using genetic science, which should be helpful to breeders in improving the quantitative and qualitative traits of livestock meat.

## Methods

### Dataset

Gene expression profiles of skeletal muscle from cattle, sheep and pig related to accession numbers E-GEOD-44,030, E-GEOD-23,563 and E-GEOD-38,518 respectively were obtained from ArrayExpress, a functional genomics data repository (https://www.ebi.ac.uk/arrayexpress) [10]. Samples obtained from longissimus muscle of Wagyu x Hereford and Piemontese x Hereford cattle, Ujomqin sheep and Pietrain pigs were used in this study. Data available in the GSE44030 dataset (20 samples, bovine skeletal muscle), were analyzed for gene expression at various time points, from 60 days postconception to 3 months of age. The GSE23563 dataset included data from 40 sheep skeletal muscle samples, with muscle development time points at 70, 85, 100, 120, 135 days postconception, birth, and 1 month and 2 months of postnatal life. Additionally, the GSE38518 dataset included 24 biological samples of porcine skeletal muscle collected at various stages of development, including 35, 63, and 91 days postconception, as well as samples from adult animals. For each species, two key stages of myogenesis were examined, in cattle 135 and 280, in sheep 70 and 120 dpc and in pigs 63 and 91 dpc.

### Differentially expressed genes

LIMMA package (Benjamini and Hochberg false discovery rate method) was used to identify differentially expressed genes (DEGs) in microarray experiments [11]. Two DEG detection criteria were found to be significant (|Fold Change| > 2 and adjusted p-Value < 0.05). Identifying genes that are differentially expressed under different experimental conditions is a typical analytical problem in genomic studies [12]. After analysing the differences in gene expression, common genes between cattle, sheep and pigs were identified using a Venn diagram (http://bioinformatics.psb.ugent.be/webtools/Venn).

### Gene ontology and kyoto encyclopedia of genes and genomes pathways

The DAVID database version 2021 [13] was used for annotation, visualization, integrated discovery, examination of GO and pathway enrichment, as well as identification of molecular function, cellular component, biological process, and KEGG pathways [14]. Checking whether a protein is annotated to GO terms or KEGG pathway can quickly reveal their relationship [15]. Following the identification of DEGs, the DAVID was used to assess the ontology of genes and pathways associated with myogenesis (Benjamini in DAVID requests adjusted p-values by using the linear step-up method of Benjamini and Hochberg [16], which was used to identify statistically significant GO Terms and KEGG pathway).

### Protein-protein interaction (PPI)

The PPI network was constructed with a STRING database (version 11.5) to detect interactions between DEGs products– proteins, and to find their different expression patterns, which are common in cattle, sheep and pigs. Protein-protein interaction is important in determining the function of target protein [16]. STRING interaction coefficients of 0.15 and above were evaluated. Modules were identified using the ClusterONE plugin of the Cytoscape software (version 3.9.1) [17]. Using a neighborhood expansion method, this plugin can detect associated proteins (modules). Hub genes were identified using CytoNCA plugin of Cytoscape software [18]. The criteria used to identify these genes were: closeness centrality, degree centrality, betweenness centrality.

### Identification miRNA and transcription factors

To identify microRNA (miRNAs) for hub genes, we employed miRWALK (http://mirwalk.umm.uni-heidelberg.de/). To detect transcription factors (TFs), we used TF2DNA (https://www.fiserlab.org/tf2dna_db/index.html) database. Additionally, we used FFLtool, a web-based platform (http://bioinfo.life.hust.edu.cn/FFLtool#!/), specifically designed to analyze Feed Forward Loop (FFL) regulatory motifs involving transcription factors, miRNAs, and genes. FFLs have the ability to regulate a multitude of target genes, contributing to the orchestration of spatio-temporal regulation and noise buffering, thus playing a crucial role in various biological processes and diseases [19].

## Results

### Differentially expressed genes

**Fig. 1:**
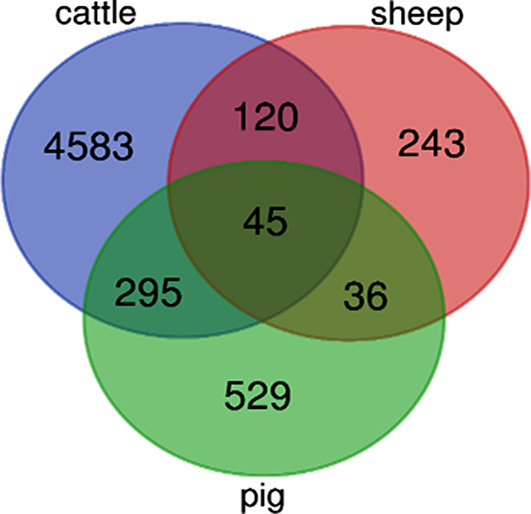
Venn diagram showing the number of genes shared by cattle, sheep and pigs

**Fig. 2:**
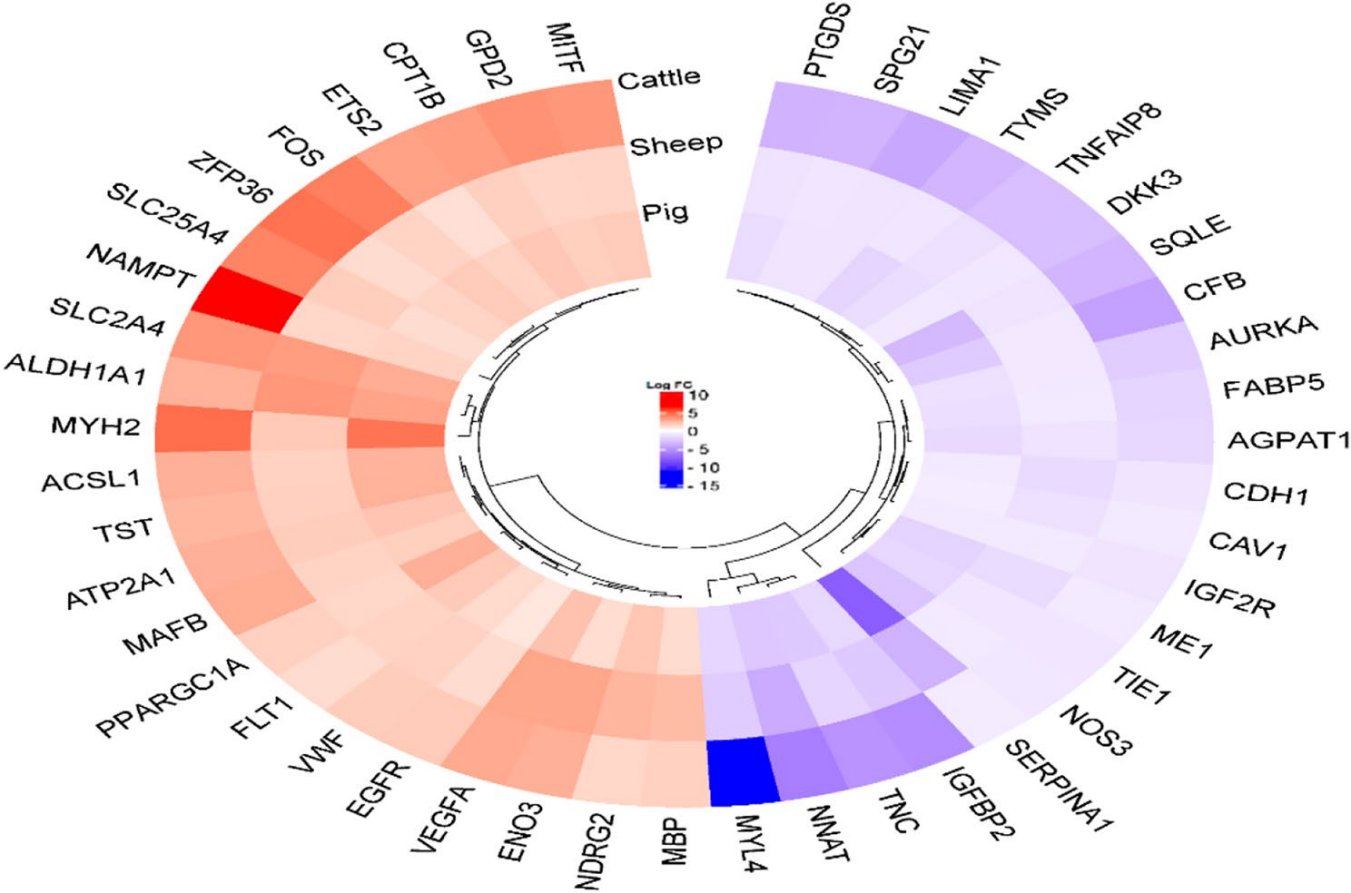
Myogenesis-related genes shared between three species

The results of the LIMMA analysis showed that in cattle 5043 genes were significantly altered between the 135 and 280 dpc, with 2574 genes upregulated and 2469 genes downregulated. In sheep, 444 DEGs were identified between the 70 and 120 dpc, with 253 genes upregulated and 191 genes downregulated. In pigs, 905 genes differed significantly between the 63 and 91 dpc stages, with 503 genes upregulated and 402 genes downregulated. A Venn diagram was used to identify genes that were common to cattle, sheep, and pigs revealing 45 genes related to myogenesis (Fig. 1), which are listed in Fig. 2 (Additional file 1, Tables S1 to S4).

### Gene ontology and kyoto encyclopedia of genes and genomes pathways

**Fig. 3:**
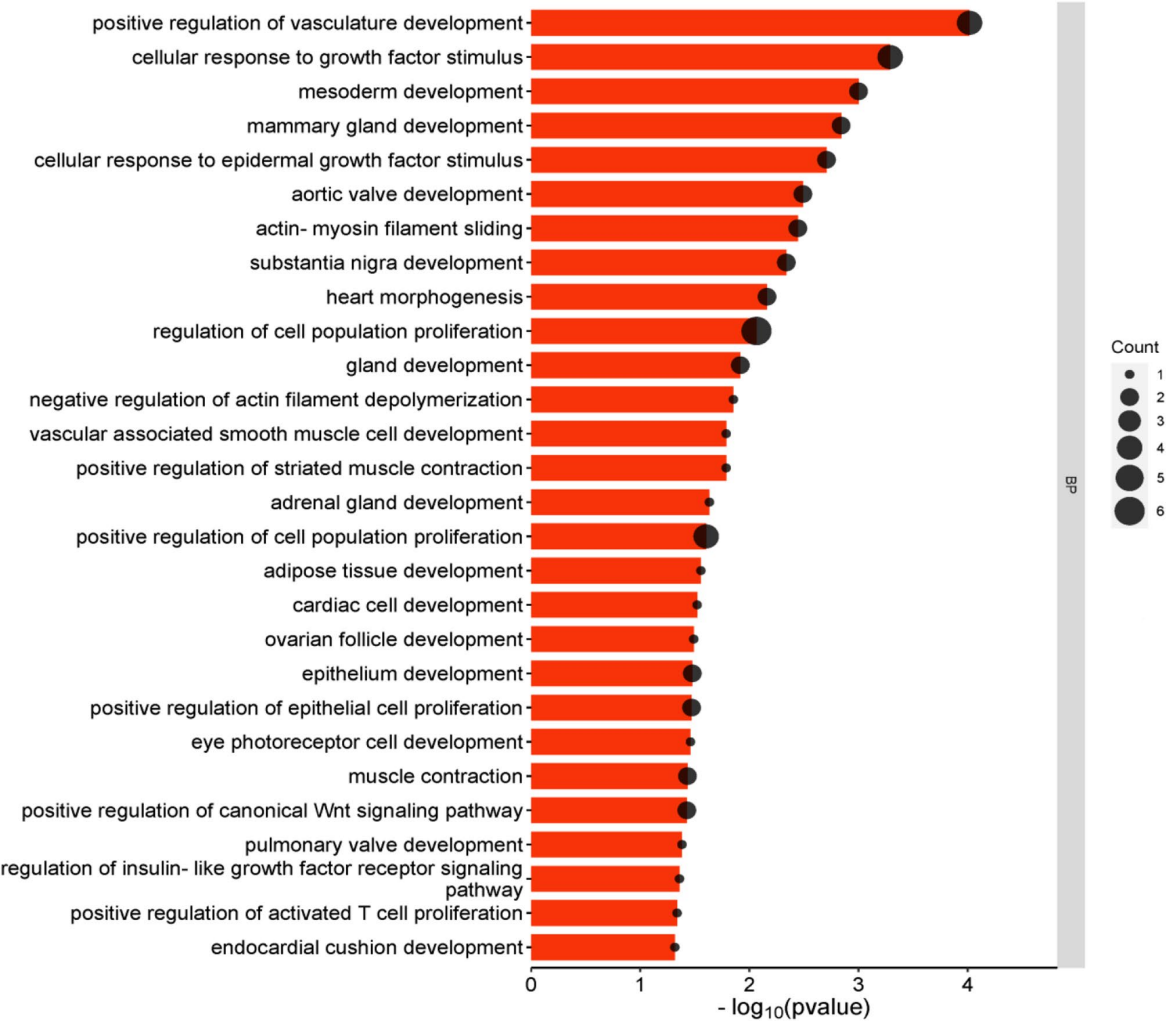
Biological processes related to myogenesis with genes common to cattle, sheep and pigs

Gene Ontology (GO) analysis using the DAVID online databse revealed that in cattle, DEGs were involved in 20 biological processes (BP) with a p-value ≤ 0.05. BP associated with skeletal muscle myogenesis are shown in Additional file 1, Table S5. GO analysis for sheep showed the involvement of DEGs in 71 biological processes with p-value ≤ 0.05. The most relevant BP with skeletal muscle myogenesis are listed in Additional file 1, Table S6. Finally, porcine skeletal muscle-specific DEGs were examined revealing 64 biological processes in which genes were involved in (with p-value ≤ 0.05). The most important ones involved in the myogenesis process are presented in Additional file 1, Table S7. Next, 45 genes common to the three species were analysed. The major common biological processes involved in skeletal muscle myogenesis are presented in Fig. 3.

**Fig. 4:**
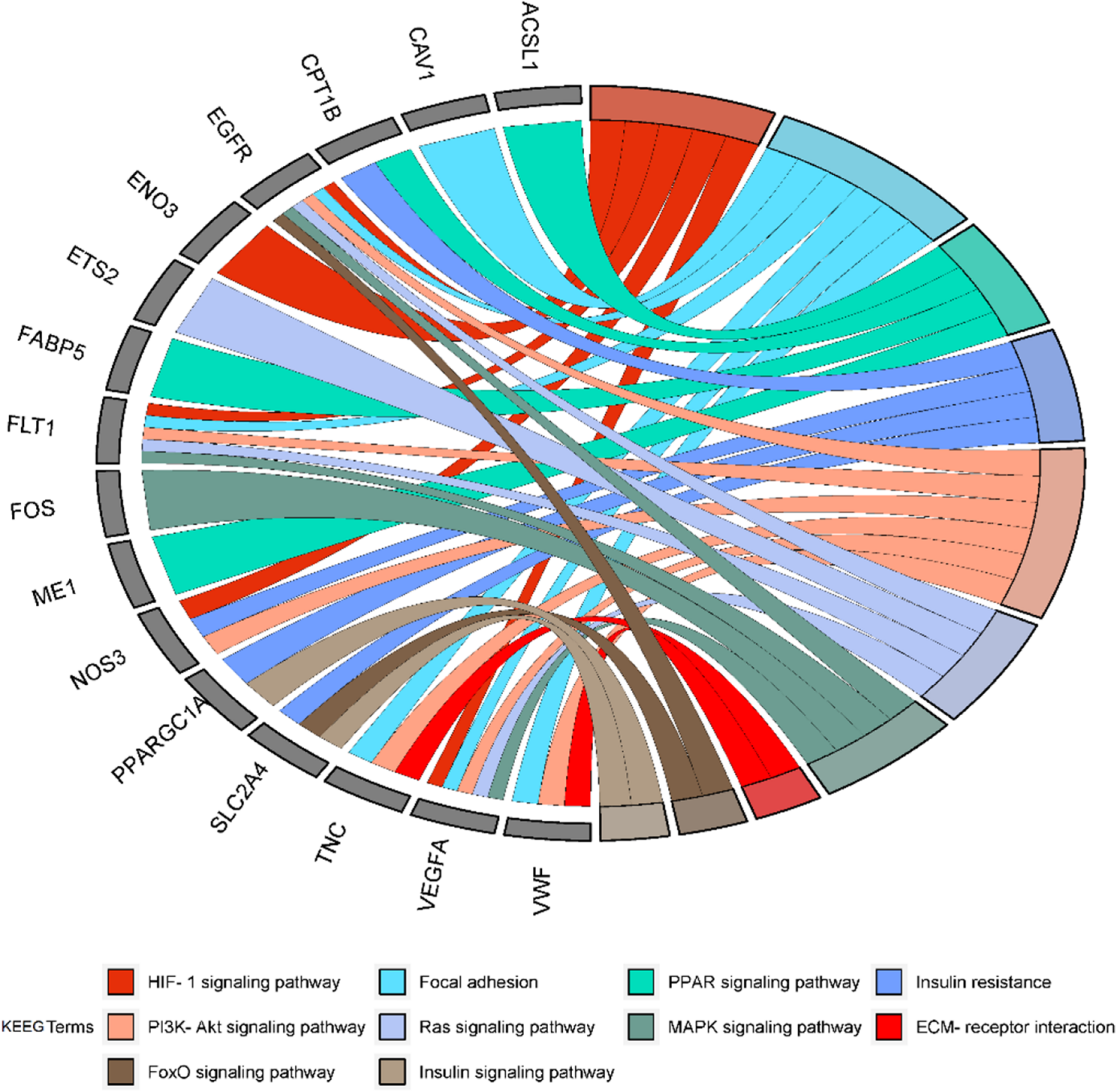
KEGG pathways with DEGs common to cattle, sheep and pigs

The DEGs were then analysed in the KEGG database to find myogenesis-specific pathways. The analysis of bovine DEGs showed that the genes were related to 31 pathways (p-value ≤ 0.05). The major pathways are shown in Additional file 1, Table S8. For sheep, 61 KEGG pathways were related to the differentially expressed genes (p-value ≤ 0.05). Important KEGG pathways related to myogenesis are listed in Additional file 1, Table S9. In pigs, the result of KEGG pathway analysis showed 63 pathways (p-value ≤ 0.05) associated with DEGs. The most significant KEGG pathways are listed in Additional file 1, Table S10. KEGG pathway analysis was then performed for common DEGs between cattle, sheep and pigs. The major common KEGG pathways related to skeletal muscle myogenesis are shown in Fig. 4.

### Protein-protein interaction

**Fig. 5:**
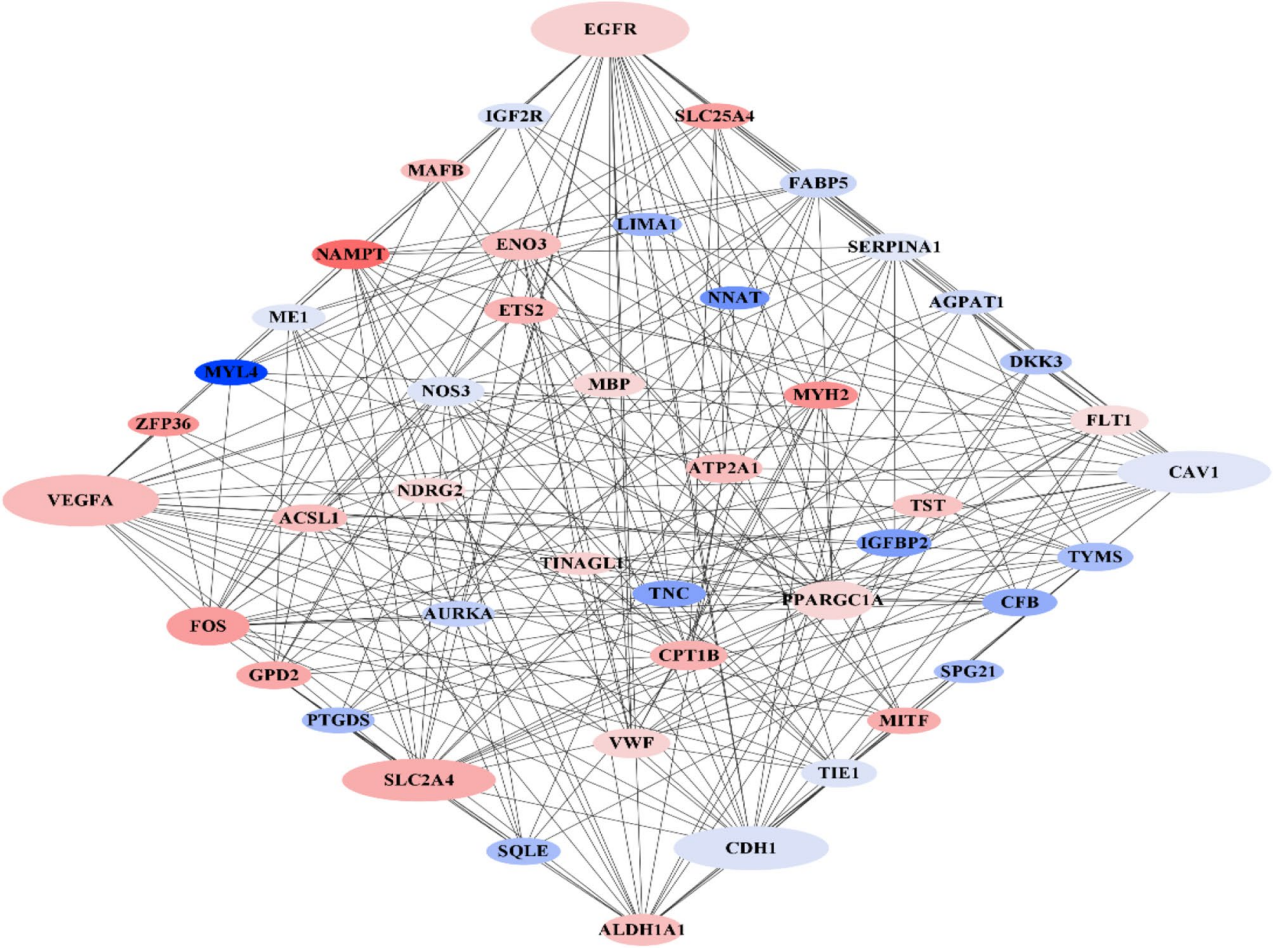
Protein-Protein Interaction network of genes common to cattle, sheep, and pigs. Red - upregulated differentially expressed genes (DEGs); blue - downregulated DEGs. The size of the node indicates the degree of centrality. Larger node size indicates a greater role in skeletal muscle myogenesis

After identifying the common genes, they were entered into the STRING database to obtain network statistics and the network image, and to indicate the role of the genes in the gene network, then the STRING output was entered into the Cytoscape software. The PPI network was created to identify the modules and hub genes. Hub genes (*EGFR*, *VEGFA*, *CDH1*, *CAV1*, and *SLC2A4*) were reported in cattle, sheep, and pig (Fig. 5; Table 1).

**Table 1:** Hub genes related to myogenesis common to cattle, sheep, and pigs (degree higher than 20; Cytoscape software)

Genes	Degree	Module	Betweenness	Closeness
*EGFR*	25	A	148.1969	0.6984127
*VEGFA*	23	A, C	136.26292	0.6769231
*CDH1*	21	A	142.25339	0.6567164
*CAV1*	20	A	148.76964	0.6376812
*SLC2A4*	20	-	127.870926	0.64705884

### Identification of network modules

The three most relevant modules were identified using the ClusterONE plugin. Module A contained 22 nodes and 105 edges. In this module, the *EGFR* gene had the highest degree with 25 edges. Module B had 20 nodes and 86 edges. The *SLC2A4* gene had the highest degree in this module, with 20 edges. Module C contained 20 nodes and 95 edges. The *EGFR* gene has the highest degree in this module with 25 edges (Table 2).

### GO and KEGG pathway analysis for modules

The genes in each module were analysed for GO and KEGG pathways with p-value ≤ 0.05. The results of the analysis in module A showed that genes with differential expression were involved in 18 BP and 22 KEGG pathways. In the case of module B, GO and the pathway results showed that DEGs in this module were related to 1 BP and 8 KEGG pathways. The analysis for module C showed their involvment in 21 BP and 20 KEGG pathways. BP and KEGG pathways related to skeletal muscle myogenesis are shown in Table 2.

**Table 2:** Characteristics of key modules in the PPI network common to cattle, sheep, and pigs

Module	Genes	P-value	GO terms	KEGG pathways
A	*TNC, PPARGC1A, CFB, VEGFA, DKK3, CAV1, SERPINA1, VWF, MITF, NOS3, ETS2, FOS, TIE1, PTGDS, FLT1, AURKA, MBP, IGF2R, CDH1, IGFBP2, EGFR, TINAGL1*	1.315*106	GO:0043406 ~ positive regulation of MAP kinase activity (FLT1, EGFR, VEGFA); GO:0048010 ~ vascular endothelial growth factor receptor signaling pathway (FLT1, VEGFA);	bta04510:Focal adhesion (FLT1, VWF, CAV1, TNC, EGFR, VEGFA); bta04151:PI3K-Akt signaling pathway (FLT1, VWF, NOS3, TNC, EGFR, VEGFA); bta04066:HIF-1 signaling pathway (FLT1, NOS3, EGFR, VEGFA); bta04015:Rap1 signaling pathway (FLT1, CDH1, EGFR, VEGFA); bta04014: Ras signaling pathway (FLT1, EGFR, ETS2, VEGFA); bta04010:MAPK signaling pathway (FLT1, FOS, EGFR, VEGFA)
B	*SPG21, PPARGC1A, MYH2, GPD2, MYL4, ATP2A1, TYMS, SLC2A4, NOS3, ENO3, NAMPT, AGPAT1, TST, SLC25A4, FABP5, CPT1B, ACSL1, ME, ALDH1A1, SQLE*	5.369*106		bta03320:PPAR signaling pathway (FABP5, ACSL1, ME1, CPT1B); bta04931: Insulin resistance (NOS3, SLC2A4, CPT1B, PPARGC1A); bta04152:AMPK signaling pathway (SLC2A4, CPT1B, PPARGC1A)
C	*PPARGC1A, CFB, VEGFA, CAV1, SERPINA1, VWF, MAFB, MITF, NOS3, ETS2, FOS, ZFP36, TIE1, FLT1, AURKA, MBP, IGF2R, CDH1, IGFBP2*	1.626*105	GO:0043406 ~ positive regulation of MAP kinase activity (FLT1, EGFR, VEGFA); GO:0048010 ~ vascular endothelial growth factor receptor signaling pathway (FLT1, VEGFA)	bta04510:Focal adhesion (FLT1, VWF, CAV1, EGFR, VEGFA); bta04066:HIF-1 signaling pathway (FLT1, NOS3, EGFR, VEGFA); bta04151:PI3K-Akt signaling pathway (FLT1, VWF, NOS3, EGFR, VEGFA); bta04015:Rap1 signaling pathway (FLT1, CDH1, EGFR, VEGFA); bta04010: MAPK signaling pathway (FLT1, FOS, EGFR, VEGFA)

### Interaction of hub genes - enrichment analysis

**Fig. 6:**
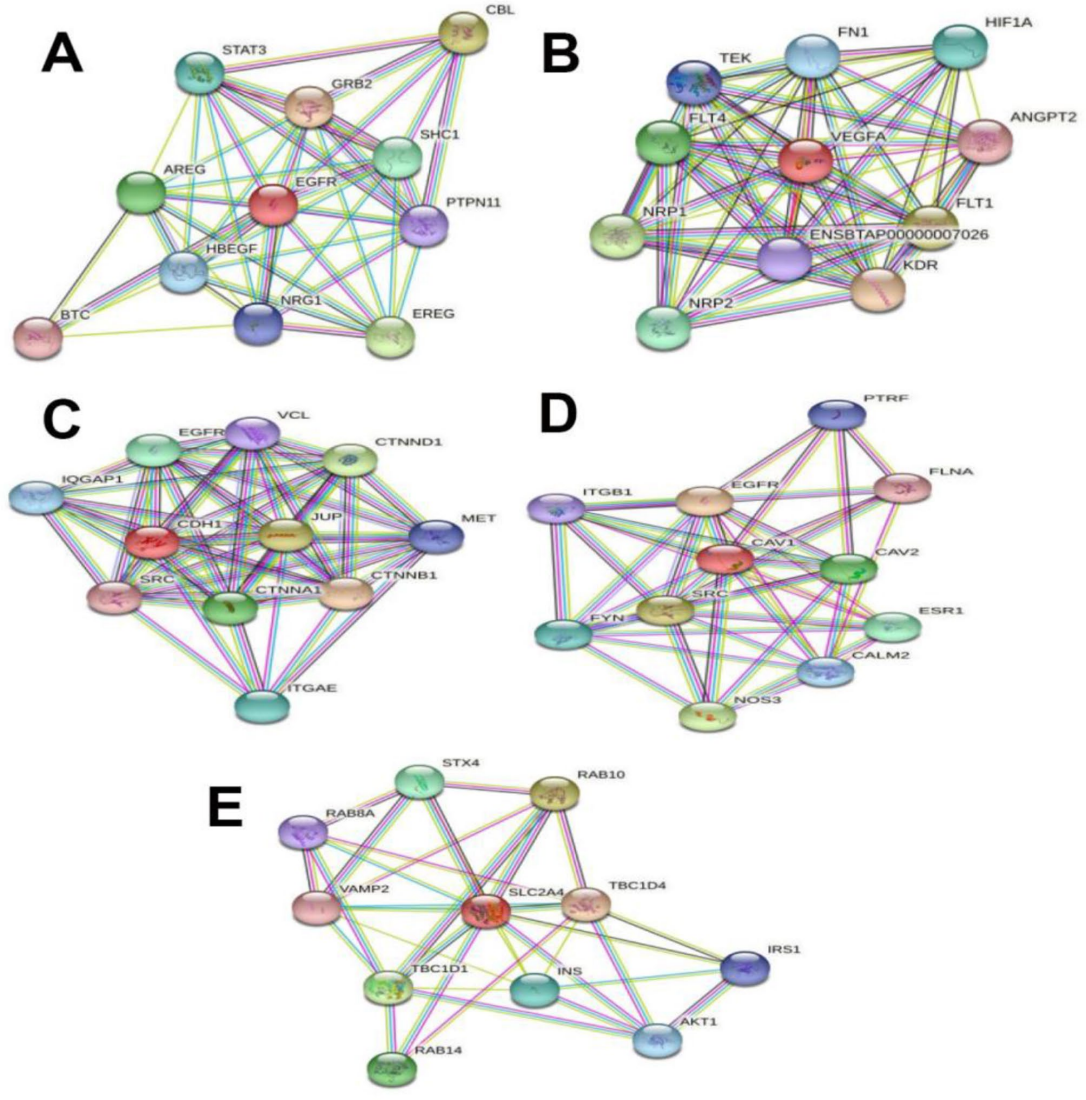
Proteinprotein interaction network of hub and relevant genes (STRING). **A**, **B**, **C**, **D** and **E** for EGFR, VEGFA, CDH1, CAV1 and SLC2A4 networks, respectively

After identifying the hub genes (Table 1) a relevance network between each hub gene and other genes was prepared using the STRING tool, showing their links to myogenesis-related genes (Fig. 6).

Subsequently, GO and KEGG pathway analysis was performed with the hub genes and the genes that interacted with them. The most important ones, related to myogenesis, are listed in Table 3.

**Table 3:** GO annotation and KEGG enrichment of genes interacting with hub genes

Hub gene	Genes	GO terms	KEGG pathways
*EGFR*	*AREG, BTC, CBL, EGFR, EREG, GRB2*	GO:0007173 ~ Epidermal growth factor receptor signaling pathway, GO:0007176 ~ Regulation of epidermal growth factor-activated receptor activity, GO:0045741 ~ Positive regulation of epidermal growth factor-activated receptor activity, GO:0007275 ~ Multicellular organism development, GO:0008286 ~ Insulin receptor signaling pathway	bta04014: Ras signaling pathway, bta04010: MAPK signaling pathway, bta04151: PI3K-Akt signaling pathway, bta04068: FoxO signaling pathway, bta04910: Insulin signaling pathway, bta04510: Focal adhesion
*VEGFA*	*ANGPT2, ENSBTAP00000007026, FLT1, FLT4, FN1, HIF1A, KDR, NRP1, NRP2, TEK, VEGFA*	GO:0038084 ~ Vascular endothelial growth factor signaling pathway, GO:0048010 ~ Vascular endothelial growth factor receptor signaling pathway	bta04151: PI3K-Akt signaling pathway, bta04015: Rap1 signaling pathway, bta04014: Ras signaling pathway, bta04010: MAPK signaling pathway, bta04066: HIF-1 signaling pathway, bta04510: Focal adhesion
*CDH1*	*CDH1, CTNNA1, CTNNB1, CTNND1, EGFR, IQGAP1, ITGAE, JUP, MET, SRC, VCL*	GO:0071363 ~ Cellular response to growth factor stimulus	bta04015:Rap1 signaling pathway, bta04510: Focal adhesion
*CAV1*	*CALM2, CAV1, CAV2, EGFR, ESR1, FLNA, FYN, ITGB1*	-	bta04510:Focal adhesion, bta04015: Rap1 signaling pathway, bta04014: Ras signaling pathway, bta04151: PI3K-Akt signaling pathway, bta04066: HIF-1 signaling pathway
*SLC2A4*	*AKT1, INS, IRS1, RAB10, RAB14, RAB8A, SLC2A4, STX4, TBC1D1, TBC1D4, VAMP2*	-	bta04152: AMPK signaling pathway, bta04931: Insulin resistance, bta04068: FoxO signaling pathway, bta04910: Insulin signaling pathway, bta04911: Insulin secretion

### Identification of miRNA-TFs-hub genes interactions

**Fig. 7:**
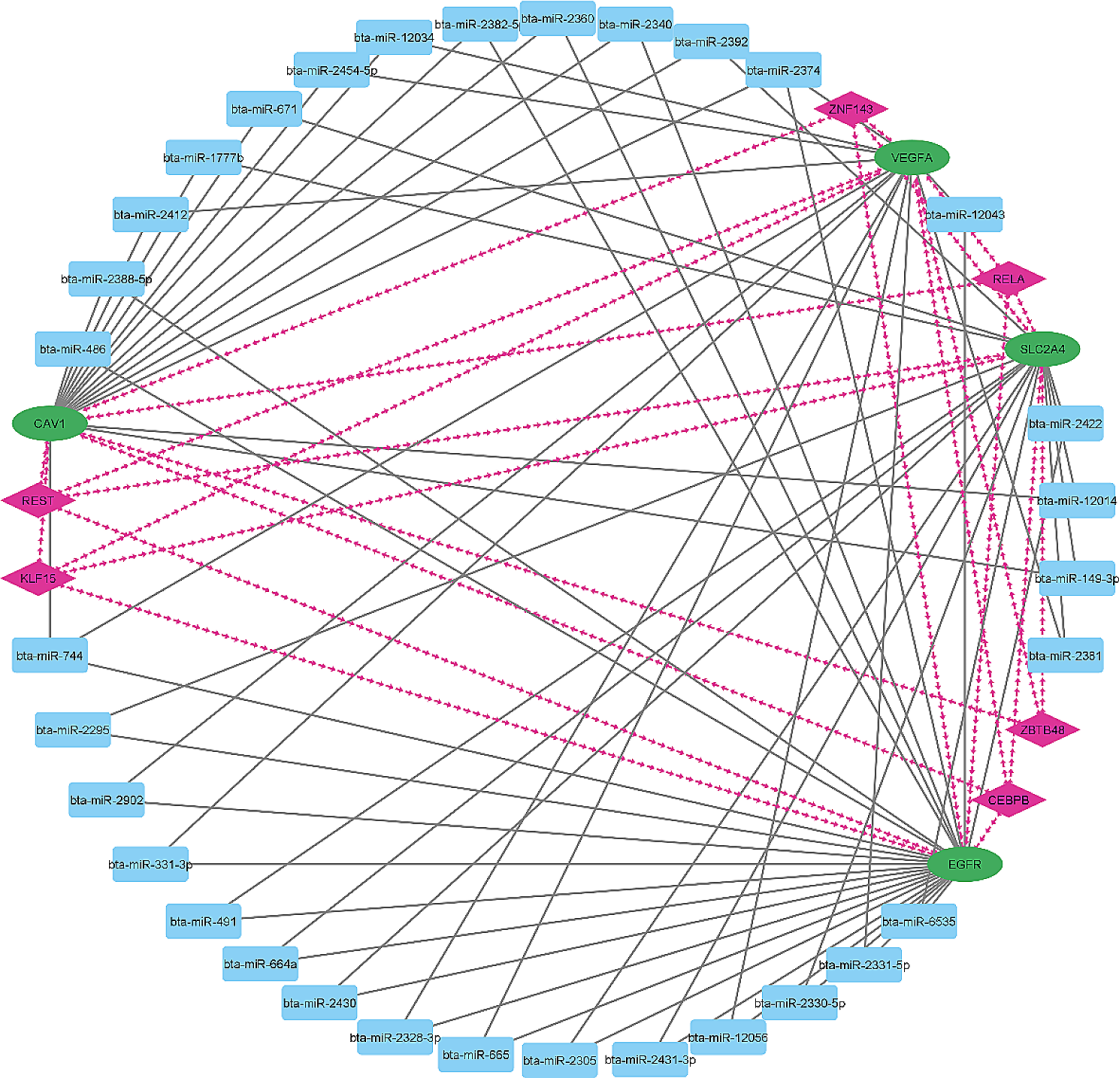
MiRNA-TFs-hub genes interactions

In this study, we attempt to uncover interactions between miRNAs, hub genes and TFs. Through our analysis, we successfully identified several TFs, specifically *CEBPB*, *KLF15*, *RELA*, *ZNF143*, *ZBTB48*, and *REST*, which play a regulatory role in controlling the expression of hub genes. Furthermore, our investigation led to the discovery of a set of miRNAs, namely bta-miR-2374, and bta-miR-744, which are closely associated with hub genes, such as *CAV1*, *EGFR*, *VEGFA*, and *SLC2A4* (Fig. 7).

## Discussion

**Fig. 8:**
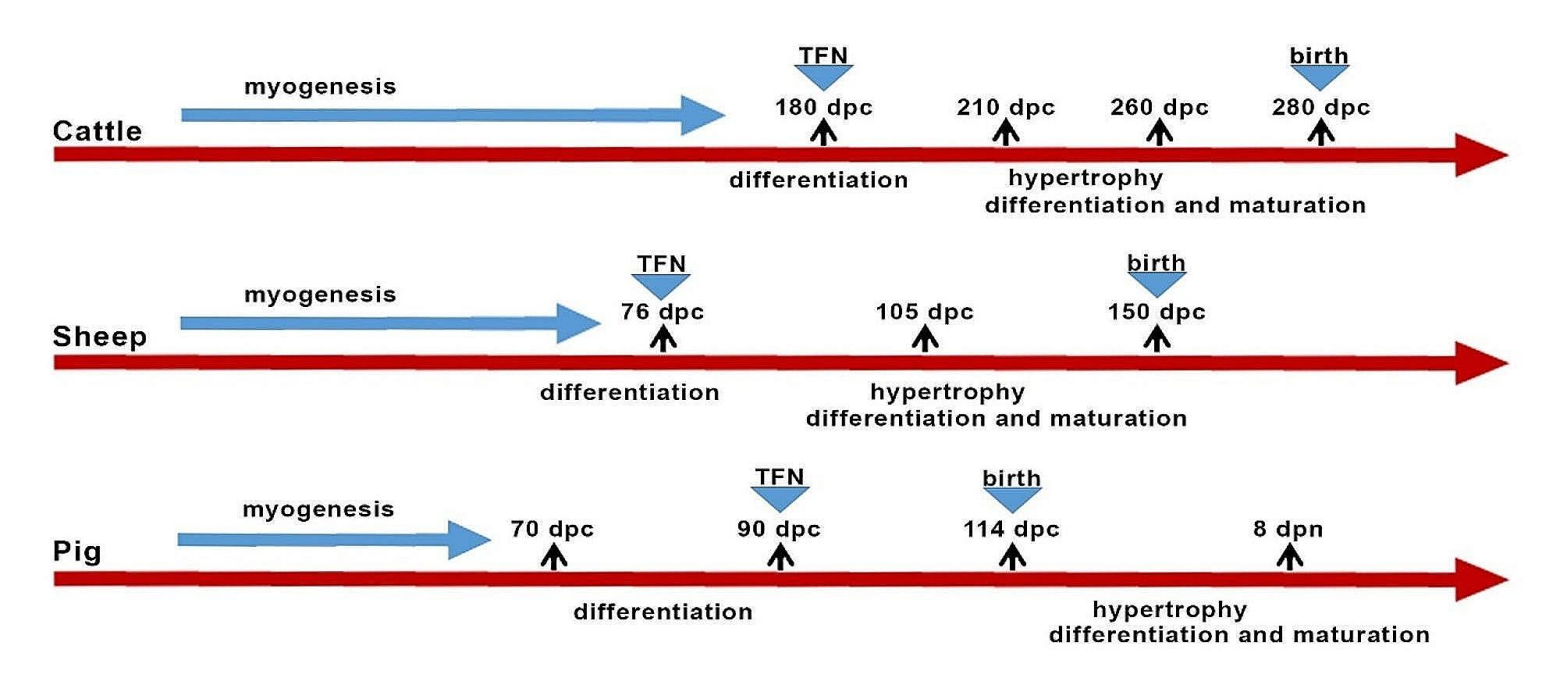
Stages of myogenesis in farm animals: cattle, sheep and pigs. Dpc - day postconception; dpn - day postnatum; TFN - total fiber number determined

The prenatal stage of myogenesis is a complex process of muscle development in animals. It includes myogenesis, fibrogenesis and adipogenesis [20]. Prenatal skeletal muscle development is a complex process that is critical for postnatal growth [21]. Skeletal muscle differentiation occurs at approximately 76 days postconception (dpc) in sheep [9, 22, 23], 180 dpc in cattle [9, 23–25], and 70–90 dpc in pigs [9, 23, 25, 26]. Muscle fiber maturation occurs in late fetal life at almost 105 days in sheep [9], 210 days in cattle [9], and 114 days in pigs [25]. After differentiation and fusion into myotubes the total fibre number (TFN) is fixed, followed by contractile and metabolic maturity [9, 22, 23, 25, 26] (Fig. 8).

The present study was performed to identify the network of genes involved in skeletal muscle myogenesis for the livestock species, such as cattle, sheep and pigs. The results revealed the total of 5043, 444 and 905 genes differentially expressed during prenatal myogenesis in cattle, sheep and pig, respectively (Fig. 1). Through the use of high-throughput functional annotation, biological process and pathway analysis, our understanding of the skeletal muscle myogenesis and its impact on muscle growth is increasing.

### Myogenesis-related biological processes - common genes

Myogenesis is a necessary process and a prerequisite for skeletal muscle development and maturation, so specifying the gene interaction modules, biological process and the pathways of the modules related to this stage seems to be a good approach for better understanding of this process. Two processes of those listed in the table of modules related to myogenesis deserve special attention: Mitogen-activated protein kinase and Vascular endothelial growth factor-activated receptor activity (Table 2). Mitogen-activated protein kinases are protein kinases that autophosphorylate their own or substrate serine and threonine residues to activate or deactivate the target [27]. MAPK kinase kinases (TAK1, MEKK4, and ASK1) recruit the MAPK kinases (MKK3 and MKK6) which ultimately phosphorylate p38MAPK. Two scaffold proteins, JLP and BNIP-2, are involved in bridging p38MAPK with MKK3/6 or the Cdc42 pathway and in turn promote myogenesis [28]. Proliferation, differentiation, survival, apoptosis, and transformation are all regulated by mitogen-activated protein kinase [29]. In our research, common genes assigned to modules associated with the positive regulation of MAP kinase activity were identified and are shown in Table 2. In modules A and C, *FLT1*, *EGFR*, and *VEGFA* genes are shown to be involved in MAP kinase activity biological process and showe a similar trend of expression difference, common to the three livestock species. In our study, *EGFR* and *VEGFA* were identified as hub genes. The vascular endothelial growth factor receptor (VEGFR) signalling pathway is a chain of chemical signals that begins with the binding of a ligand to a VEGFR on the surface of a target cell and ends with the control of a downstream cellular function. VEGFB is a protein that regulates proliferation and differentiation multiple cells [30]. PI3K/Akt/mTOR signalling pathway acts via VEGFB activation, which in turn promotes myoblast development [30]. Considering the activity of genes and their involvement in biological processes and signaling pathways, modules A and C play an important role, and the hub genes identified in this study, are strongly involved in skeletal muscle myogenesis.

### Myogenesis-related KEGG pathways - common genes

According to our findings, pathways related to myogenesis have been identified in each module (Table 2). One of them is the Rap1 signaling pathway, which is involved in cell adhesion and cell-cell junction formation [31]. Statins act indirectly on Rap1 by inhibiting the availability of non-sterol isoprenoids in muscle cells. Prenylation of the Rap1 protein is disrupted, resulting in reduced muscle cell survival (repressed protein synthesis) and autophagy. In statin-dependent myopathies, this pathway may explain muscle demage and impaired myogenesis [32]. This pathway is located in the module A and C, and the common genes involved in it are *FLT1, CDH1, EGFR*, and *VEGFA*. There are three hub genes in this pathway (Table 1).

In myoblasts, stimulation of the PI3K/Akt/mTOR signaling pathway resulted in increased protein production. On the other hand, wortmannin-mediated suppression of PI3K/Akt with and VEGFR1 knockdown prevented VEGFB-induced myoblast development [30]. By suppressing AKT signaling, high glucose levels prevented myogenic differentiation. In C2C12 myotubes, high glucose levels cause insulin resistance [33]. Myogenin is involved in the assembly of myoblasts into myotubes, an important step in myogenesis. As mentioned above, the genes *FLT1, VWF, NOS3, TNC, EGFR*, and *VEGFA* play an important role in this pathway and are located in module A and C as hub genes (Tables 1 and 2).

Peroxisome proliferator-activated receptors (PPARs) are transcription factors in the nuclear receptor family. PPARs regulates genes relinvolved in growth, metabolism, and various cell functions in the body [34]. PPAR is a positive regulator of skeletal muscle myogenesis that acts by inhibiting myostatin activity through a Gasp-1-dependent mechanism [35]. In our study, PPAR pathway was localized to module B, and according to our results, the set of genes involved includes *FABP5, ACSL1, ME1*, and *CPT1B* (Table 1). Evidence suggests that gene interactions of the above-mentioned pathways are critical for the investigated myogenesis stage.

### Myogenesis-related hub genes common to cattle, sheep, and pigs

Five hub genes specific to skeletal muscle myogenesis have been identified by analyzing a network of common genes in cattle, sheep and pigs (Fig. 5; Table 2).

The first hub gene, epidermal growth factor receptor (*EGFR*) was identified with the highest degree of linkage. Myoblast formation is known to be regulated by the *EGFR*. Inhibition of differentiation is one of the physiological actions of *EGFR* in proliferating myoblasts, and an early *EGFR* downregulation is critical for myoblast differentiation. Therefore, *EGFR* activation should prevent the induction of myoblast differentiation [36]. According to our results, *EGFR* interacts with *AREG, BTC, CBL, EREG*, and *GRB2* genes (Fig. 6). They are all involved in epidermal growth factor receptor-related processes listed in Table 3, including epidermal growth factor receptor binding, growth factor activity, and insulin receptor signaling pathway. The GPR39*/*β-arrestins/Src complex regulates *EGFR* from a mitogenic to a myogenic stimulus by giving *EGFR* the ability to activate pathways consistent with a specific myogenic signature. This mechanism would allow *EGFR* to provide a cell cycle exit signal to promote myoblast differentiation and fusion into multinucleated mature myotube [37].

One of the hub genes is vascular endothelial growth factor (*VEGF*). *VEGF* has been used to improve muscle function, increase muscular vasculature, and minimize local inflammation in Duchenne muscular dystropy [38]. *VEGF* can affect the number of satellite cells, their activation, and muscle capillary density. Due to the proximity to endothelial and satellite cells, their reciprocal effects on skeletal muscle development has been observed [39, 40] (40,41). Our analysis confirmed that *ANGPT2, ENSBTAP00000007026, FLT1, FLT4,FN1, HIF1A, KDR, NRP1, NRP2, TEK*, and *VEGFA* genes interact with *VEGF* hub gene (Fig. 6) and are involved in the processes listed in Table 3, including PI3K-Akt signaling pathway, Rap1 signaling pathway, Ras signaling pathway, MAPK signaling pathway, HIF-1 signaling pathway, and vascular endothelial growth factor signaling pathway (Table 3).

Another hub gene identified is cadherin1 (*CDH1*). Loss of *CDH1* induced muscle satellite cells to enter the cell cycle, and recurrent muscle injury reduced the pool of muscle satellite cells. Previous findings have shown that the Cdh1–FoxM1–Apc axis is an important regulator of muscle growth and regeneration [41]. According to our results, *CDH1, CTNNA1, CTNNB1, CTNND1, EGFR, IQGAP1, ITGAE, JUP, MET, SRC*, and *VCL* seem that the aforementioned genes can influence myogenesis by participating in such biological processes and pathways: Rap1 signaling pathway, focal adhesion, and cellular response to growth factor stimulus (Table 3).

Caveolin 1 (*CAV1*) a hub gene, together with the following genes *CALM2, CAV2, EGFR, ESR1, FLNA, FYN*, and *ITGB1* is directly or indirectly involved in focal adhesion, Rap1 signaling pathway, Ras signaling pathway, PI3K-Akt signaling pathway, and HIF-1 signaling pathway (Table 3). *CAV1.1* is expressed in skeletal muscle where, in addition to L-type calcium channel activity, it acts as a voltage sensor for excitation-contraction coupling (ECC) and skeletal muscle contraction [42]. *CAV1.1* has important, slightly different functions in embryonic and fetal skeletal muscle development, in addition to its critical involvement in skeletal muscle ECC [43].

Solute Carrier Family 2 Member 4 (*SLC2A4*) is the last hub gene identified in our study. Glucose transporter type 4 (*GLUT4*) is a protein encoded by the *SLC2A4* gene. *GLUT4* is stimulated by activation of Akt in PI3K-dependent mechanism, resulting in glucose uptake [44, 45]. Previous findings have shown that decreasing the expression of *STK25* gene increases the expression of *Slc2a1* and *Slc2a4* genes, resulting in insulin-facilitated glucose uptake in muscle cells [46]. Most cells require an energy source for growth and differentiation. Our results show that myogenesis can be controlled by *AKT1, INS, IRS1, RAB10, RAB14, RAB8A, SLC2A4, STX4, TBC1D1, TBC1D4*, and *VAMP2* genes involved in AMPK signaling pathway, insulin resistance, FoxO signaling pathway, insulin signaling pathway, and insulin secretion pathway (Table 3).

### Transcription factors regulating hub genes

Both TFs and miRNAs have the ability to influence gene expression. In this study, the interactions between miRNAs, TFs and hub genes were investigated to better understand the mechanisms underlying prenatal myogenesis in livestock. Some TFs that can potentially act as regulators of hub genes during skeletal muscle development were identified. CEBPB, KLF15, RELA, and REST, which we have shown can regulate some hub genes, namely *EGFR*, *SLC2A*, *CAV1* and *VEGFA* (Fig. 8), contributing to skeletal muscle regeneration and adipogenesis and indirectly affecting muscle growth. In addition, a group of miRNAs, specifically bta-miR-2374 and bta-miR-744, were found to be closely associated with hub genes such as *CAV1*, *EGFR*, *VEGFA*, and *SLC2A4* (Fig. 7). It is well-known, that *KLF15* is involved in the regulation of skeletal muscle metabolism and muscle fiber size, acting as suppressor of muscle growth [47, 48]. Several studies support the role of RELA in the NF-kB complex in muscle regeneration and remodeling. One study found that activation of the NF-kB signaling pathway involving RELA is critical for muscle regeneration after injury. Meanwhile, another study observed that inhibition of NF-kB signaling involving RELA leads to impaired muscle regeneration in mice [49, 50]. RE1-silencing transcription factor (REST), serves as a key transcriptional repressor in shaping the development of the nervous system. Its influence extends beyond neurons, as it also plays an important role in directing the proper differentiation of various cell types, including hematopoietic stem cells differentiation into erythrocytes and myogenic regulatory factors critical for muscle cell differentiation [51, 52].

ZNF143 and ZBTB48, two TFs with potential effects on muscle cell development [53, 54], act through different mechanisms and we show that they can regulate *EGFR*, *SLC2A*, *CAV1* and *VEGFA*. ZNF143, known to regulate genes important for cell cycle and metabolism, is speculated to contribute to muscle cell development [53]. However, the exact mechanism underlying the effect of ZNF143 on muscle cell development remains elusive. On the other hand, ZBTB48 is a protein involved in maintaining telomere length, a critical factor for cell division and aging [54]. Although its role in myogenesis is not fully understood, it is suggested that ZBTB48 may indirectly influence muscle cell development and aging through its involvement in telomere maintenance [55]. In summary, both ZNF143 and ZBTB48 potentially affect muscle cell development, each through a different pathway, and further research is needed to fully understand their roles in this process. By constructing a regulatory network of TF-miRNA-hub genes, we have attempted to depict the involvement/relevance of the genes described above in the complex network of relationships underlying skeletal muscle development, hypertrophy and maturation in livestock.

## Conclusions

In study of gene expression profiles of bovine, ovine and porcine muscle tissue, hub genes, biological processes and pathways related to myogenesis were identified. Based on the results of the network analysis drawn by the common genes, it seems that the five described hub genes have the greatest regulatory influence on the process of myogenesis. Therefore, the results of this study may provide additional information for a better understanding of the relationship between transcription factors, hub genes and their signaling pathways involved in prenatal myogenesis, especially in livestock, that may affect differentiation and hypertrophy and ultimately meat production. Among the large number of genes and biological pathways involved in this important economic process, their introduction will help geneticists to put more emphasis on developing breeding programs to improve growth traits and meat production using related genes and biological pathways, and In less time, the desired breeding objectives will be realized.

### Electronic supplementary material

Below is the link to the electronic supplementary material.

Supplementary Material 1

## Data Availability

The data supporting the findings of this study was downloaded from “ArrayExpress, a functional genomics data repository (https://www.ebi.ac.uk/arrayexpress) [10]”.
