# Supplementary material for: Network visualization of genes involved in skeletal muscle myogenesis in livestock animals
Source: BMC Genomics. 2024 Mar 19;25:294. doi: 10.1186/s12864-024-10196-3 (PMC10953195; doi:10.1186/s12864-024-10196-3)
Supplement: Supplementary file 1 — Supplementary Material 1 [file 12864_2024_10196_MOESM1_ESM.docx]

**Table S1** Symbol of common genes identified between 3 species using Venn diagram

| Cattle, sheep, pig | AGPAT1,PPARGC1A,NDRG2,SQLE,IGFBP2,TYMS,VEGFA,EGFR,SLC25A4,ACSL1,ENO3,MBP,NNAT,ETS2,SERPINA1,TIE1,PTGDS,FOS,CAV1,ALDH1A1,SLC2A4,NOS3,GPD2,MITF,FABP5,MYH2,AURKA,ZFP36,FLT1,NAMPT,DKK3,TST,MAFB,IGF2R,ME1,MYL4,ATP2A1,LIMA1,CPT1B,VWF,TNC,SPG21,CDH1,TNFAIP8,CFB |
| --- | --- |

**Table S2** Symbol of common genes identified between cattle and sheep using Venn diagram

| Cattle, sheep | MMP2,XDH,NOS2,KCNK3,COL1A1,CA11,ABR,APOE,CDK1,PRND,FAU,MYF5,PGM1,SHH,TAC1,ACLY,OSTN,BGN,ASPN,AK1,ABHD5,GOLGB1,HBB,GNG11,MYL9,LPL,RAB28,UCP3,COL1A2,COL12A1,ATOX1,PHKG2,EZH2,CD40,TECR,SNX10,PPTC7,FAR2,PRLH,LDHA,HSPA1A,CDCA7,BUB1,F8,LSS,PDIA5,SIGIRR,RTP4,GFAP,ACTB,CD96,NOG,DAP,GPX3,KLK1,CSN2,PYGM,LPAR1,JUN,PON3,OXT,SMO,CNN1,GHR,ALDH2,MT3,MYH1,FN1,RBM38,SELP,KRT8,FHIT,IL21R,CD86,MDFI,EEF1A2,COPB2,TCF7L1,TRPM7,PGF,POSTN,PLAT,ADAMTS15,CLEC7A,PLIN5,LYZ,GAPDH,SLC16A1,CKAP4,MID1IP1,CLDN4,PLK1,DPP4,TMEM159,S100A14,PTK7,KYNU,IL15,TRIB1,CTSD,PTCH1,GRIA3,SLC7A8,ELOVL4,EEF1A1,ASPM,PPARA,UGDH,LOXL2,COL2A1,THBS2,LTF,ANPEP,NPPC,RAD51,RAB27A,BCL11A,BCAT1,NRG1,ADIPOQ |
| --- | --- |

**Table S3** Symbol of common genes identified between cattle and pig using Venn diagram

| Cattle, pig | HAUS2,STK16,SEMA4D,LHFPL2,TMEM52,C1QTNF3,GDPD5,GSTA2,DSTN,ORM1,CD82,ORC1,PTP4A2,TOMM34,TOX3,TF,NCAM1,COL7A1,COX10,ASAH1,RGS14,ACO2,HP,GOT2,CRABP1,LDLR,POLR1A,ACADVL,TNNT2,ARHGAP25,NRAP,NCF1,IL18,MGP,FKBP5,COMTD1,GPX2,PTPN4,EPB41L3,HN1,DDIT4L,PPP6R2,CDH11,SGSM3,PHYH,DNASE1L1,ALAS1,TRIP10,GADD45B,SLC22A5,ARHGDIB,5Sep,PPP1R15A,CSDC2,BGLAP,ACY1,PPP5C,ADHFE1,TMEM38A,CAPNS1,CXCL2,EML4,TTL,STAT5B,TCAP,CDC45,IER5,RNPEP,MSMO1,SGSH,ZNF263,SLC41A1,IL17B,OTC,LMCD1,NFKBIA,C1QA,ALMS1,COL11A1,PCOLCE2,FBXL4,PROCR,RNASE4,MAPK8IP1,RAP1GDS1,PTN,HSPB6,CAPN3,CCDC12,STRADB,SMAD3,ANKRD10,CACNB3,DDAH1,CHEK1,CKB,SYN1,CHD3,GDI1,TAPBP,ARPC5,MAPRE3,SCD,YDJC,TRPT1,INPP1,DUSP1,CPM,TMEM106A,GART,S100A1,KDELR3,HEBP2,DPT,RGS5,TMEM150C,PECAM1,B3GNT3,OCIAD2,TGFB3,B3GALNT1,CDO1,NLN,PREB,S1PR1,METTL22,CCDC80,CLU,NR5A2,DGAT2,CLEC1A,PLBD1,SHROOM3,ASNS,KIAA1191,FAM43A,AKT1S1,KLF15,ME3,FABP4,CDC34,NDUFA9,CTSZ,SCG5,SNAP91,SFRP1,ARHGAP27,ARNT2,FIBIN,PEBP4,GOT1,ATP5D,PAPSS2,KPNA2,PLEKHO1,PLIN2,LDHB,RYR1,PBX1,TMEM25,PPL,CHAF1A,CEBPD,TMEM170A,NGFRAP1,FYN,CCL14,MAP1A,CD200,BAG3,CCL4,ADCK2,GAP43,SREBF2,ACAN,GPT,PDK4,HABP2,PARP8,FN3K,CCDC167,BLNK,HAS3,DYRK3,CRABP2,ACTR3B,ASL,ANGPTL4,ACOT13,AMFR,RHOU,DMXL2,FAM57A,BLVRA,FXR2,PIP4K2A,ARHGAP1,FCHO1,NAAA,PLSCR1,SLC25A11,NFS1,PDE4B,DLGAP5,SGCD,IL4R,TMEM120A,CDKN2C,MAPK8IP3,KLF13,PARP9,ACTG1,KCNJ8,ITIH4,INSIG1,SATB1,PRCP,RNF135,TMOD4,AK3,FBP2,FASN,CAMK1,SLC44A2,ATF3,UCHL1,PNMT,HSPB3,THBS3,IDH2,SEC31B,ASF1B,CAMK2D,TNNI3,ISOC2,JAG1,AFP,FCN2,ARHGAP28,FAM110A,ZNF281,PPP1R3B,CALR,LTB,TTC39C,PCK1,CHST14,TSPAN33,SMPDL3A,NDOR1,FKBP1B,NANP,TYRO3,ARNTL,S100A11,ACSS2,ROCK1,CD34,RXRG,KDR,MYLIP,MGLL,ATP1A2,RBP4,SDPR,HOMER2,DNMT1,MB,TSPO,POLE2,SCN2A,KHDRBS3,2Mar,PPIF,SLC16A3,ITIH2,ADK,CA2,PAPSS1,COX7A1,SERPINE1,PLLP,ALS2CL,HMMR,ENPP1,RPUSD4,LPCAT3,RAMP2,ANKRD1,ALDOB,PTGS1,HGSNAT,FDPS,NDUFS7,ATP5G1 |
| --- | --- |

**Table S4** Symbol of common genes identified between pig and sheep using Venn diagram

| Pig, sheep | TEK,CD93,PSMB8,APOBEC3F,BHLHE40,PEG3,SHTN1,IRF1,TAP1,EGR1,CLDN7,PTGFR,TM7SF2,CAPG,TTR,HSD11B1,COL5A1,CDH5,ARL4A,KIF2A,JADE2,MX1,LOX,AHSG,EPAS1,AFF1,AKAP7,SQSTM1,ATP1B1,PPARG,FBLN1,ILF3,CLCN5,PDLIM3,CEBPB,ATIC |
| --- | --- |

**Table S5** Biological processes related to skeletal muscle myogenesis with differentially expressed genes for cattle

| Gene Set | Description | p-Value | Count |
| --- | --- | --- | --- |
| GO:0042127 | regulation of cell proliferation | 3.91E-04 | 61 |
| GO:0051301 | cell division | 8.88E-04 | 61 |
| GO:0002076 | osteoblast development | 0.004897 | 10 |

BP – biological process; p-Value ≤ 0.05.

**Table S6** Biological processes related to skeletal muscle myogenesis with differentially expressed genes for sheep

| Gene Set | Description | p-Value | Count |
| --- | --- | --- | --- |
| GO:0048662 | negative regulation of smooth muscle cell proliferation | 3.10E-05 | 6 |
| GO:0048146 | positive regulation of fibroblast proliferation | 0.002347 | 6 |
| GO:0001937 | negative regulation of endothelial cell proliferation | 0.018758 | 4 |
| GO:0061053 | somite development | 0.026039 | 3 |

BP – biological process; p-Value ≤ 0.05.

**Table S7** Biological processes related to skeletal muscle myogenesis with differentially expressed genes for pigs

| Gene Set | Description | p-Value | Count |
| --- | --- | --- | --- |
| GO:0016202 | regulation of striated muscle tissue development | 0.0170723 | 3 |
| GO:0030199 | collagen fibril organization | 0.0075218 | 6 |
| GO:0032024 | positive regulation of insulin secretion | 0.0390140 | 4 |

BP – biological process; p-Value ≤ 0.05.

**Table S8** KEGG pathways related to skeletal muscle myogenesis with differentially expressed genes for cattle

| gene Set | description | p-Value | Count |
| --- | --- | --- | --- |
| bta04151 | PI3K-Akt signaling pathway | 6.31E-06 | 127 |
| bta04510 | Focal adhesion | 3.30E-05 | 81 |
| bta03320 | PPAR signaling pathway | 5.85E-04 | 32 |
| bta045120 | ECM-receptor interaction | 7.78E-04 | 37 |
| bta04010 | MAPK signaling pathway | 00.001061 | 89 |
| bta04152: | AMPK signaling pathway: | 0.001609 | 47 |
| bta04014 | Ras signaling pathway | 0.002692 | 81 |
| bta04015 | Rap1 signaling pathway | 0.00381 | 71 |

KEGG pathway – [Kyoto Encyclopedia of Genes and Genomes pathways](https://www.genome.jp/kegg/); p-Value ≤ 0.05.

**Table S9** KEGG pathway related to skeletal muscle myogenesis with differentially expressed genes for sheep

| gene Set | description | p-Value | Count |
| --- | --- | --- | --- |
| oas04510 | Focal adhesion | 2.11E-06 | 23 |
| oas04151 | PI3K-Akt signaling pathway | 1.78E-05 | 29 |
| oas04066 | HIF-1 signaling pathway | 4.62E-04 | 12 |
| oas04512 | ECM-receptor interaction | 8.34E-04 | 11 |
| oas04917 | Prolactin signaling pathway | 0.021257 | 8 |
| oas04015 | Rap1 signaling pathway: | 0.02308 | 14 |
| oas03320 | PPAR signaling pathway | 0.029791 | 7 |
| oas04931 | Insulin resistance | 0.034662 | 9 |

KEGG pathway – [Kyoto Encyclopedia of Genes and Genomes pathways](https://www.genome.jp/kegg/); p-Value ≤ 0.05.

**Table S10** KEGG pathway related to skeletal muscle myogenesis with differentially expressed genes for pigs

| gene Set | description | p-Value | Count |
| --- | --- | --- | --- |
| ssc03320 | PPAR signaling pathway | 0.001358 | 12 |
| ssc04510 | Focal adhesion | 0.003394 | 22 |
| ssc04068 | FoxO signaling pathway | 0.008997 | 16 |
| ssc04512 | ECM-receptor interaction | 0.016944 | 11 |
| ssc04066 | HIF-1 signaling pathway | 0.022622 | 12 |
| ssc04152 | AMPK signaling pathway | 0.035612 | 13 |

KEGG pathway – [Kyoto Encyclopedia of Genes and Genomes pathways](https://www.genome.jp/kegg/); p-Value ≤ 0.05.
